# Effect of neurokinin-1-receptor blockage on fracture healing in rats

**DOI:** 10.1038/s41598-019-46278-6

**Published:** 2019-07-05

**Authors:** Martijn Hofman, Frederik Rabenschlag, Hagen Andruszkow, Julia Andruszkow, Diana Möckel, Twan Lammers, Aneta Kolejewska, Philipp Kobbe, Johannes Greven, Michel (Paul Johan) Teuben, Martijn Poeze, Frank Hildebrand

**Affiliations:** 1 Department of Orthopedic Trauma and Reconstructive Surgery, University of Aachen Medical Center, Aachen, Germany; 2 Institute of Pathology, University of Aachen Medical Center, Aachen, Germany; 3 Institute for Experimental Molecular Imaging, Center for Biohybrid Medical Systems, University of Aachen Medical Center, Aachen, Germany; 4 Department of Trauma and Harald Tscherne Laboratory, University Hospital Zurich, Zurich, Switzerland; 5 Department of Surgery, Division of Traumasurgery, Maastricht University Medical Center, Maastricht, The Netherlands

**Keywords:** Preclinical research, Experimental models of disease

## Abstract

Neurologic injury and selective blockage of sensory nerve endings is associated with impaired fracture healing, however, the role of specific neurotransmitters has not been sufficiently investigated. Our aim was to investigate the impact of specific Substance P-receptor blockage on fracture healing, since the neuropeptide Substance P has both neurogenic and osteogenic activity. After intramedullary stabilization, an isolated femur fracture was induced in 72 Sprague-Dawley rats. In the NK1-R group, the neurokinin-1-tachykinin receptor for substance P was blocked by a specific antagonist (SR140333) for the first two weeks after fracture induction. The control group only received vehicle. Gene-expression, histology, micro-computed tomography, and biomechanical tests were performed. NK1-receptor blocking suppressed osteocalcin expression at one week, collagen 1A2 expression at one and two weeks and collagen 2A1 expression at 2 weeks after fracture induction. Biomechanical testing revealed a significant reduction in maximal load to failure in the NK1-R group at 6 weeks (69.78 vs. 155.45 N, p = 0.029) and at 3 months (72.50 vs.176.33 N, p = 0.01) of fracture healing. Blocking the NK1-receptor suppresses gene expression in and reduces biomechanical strength of healing bone. Therefore, we assume a potential therapeutic relevance of Substance P in cases of disturbed fracture healing.

## Introduction

Fractures are frequently associated with either central (e.g., traumatic brain injury [TBI]) or peripheral neurological injuries, and these injuries are well known to affect fracture healing. This phenomenon has partly been explained by the close anatomical relationship between bones and nerves (e.g., innervation of periosteum with close contact between nerve endings and bone cells). It also reflects the chronological association between callus formation, bone remodelling, and the regeneration of damaged nerve endings and ingrowth into newly formed bone^1,2^. Disturbances of this re-innervation have therefore been associated with the incidence of non-unions^3^. Hukkanen and colleagues provided the first evidence for a role of nerve-mediated bone formation in fracture healing by showing that a complete denervation of a leg by sciatic nerve section had negative effects on the mechanical integrity of the bony callus after fracture^4^. Similarly, Offley and colleagues found that capsaicin-sensitive neurons contribute to cancellous bone integrity and bone homeostasis in the uninjured bone. They concluded that osteoclast numbers, osteoblast activity, bone formation, and bone strength were mediated by transmitters released from efferent sensory nerve endings in bone tissue^5^.

Further, complete peripheral nerve transection^6–8^ and selective blockade of sensory nerve endings^9^ can impair fracture healing, as demonstrated by previous studies utilizing either combined motoric, sensory, and autonomic denervation^7,8^ or complete sensory nerve ending blockade^5,9^.

The exact pathophysiologic mechanism underlying this interaction between fracture healing and nerve injuries is not known. Neuropeptides are produced by nerve endings after TBI and fractures, so neuropeptides potentially have major relevance in the process of fracture healing^1,2,10,11^. However, no precise role has been established for specific neuropeptides in bone healing^12,13^.

One neuropeptide of particular relevance to bone healing is Substance P, which shows simultaneous effects at the neurogenic and osteogenic activities. For example, Apel *et al*. showed that sensory denervation, which shut down the transmission of both calcitonin gene-related peptide (CGRP) and Substance P, resulted in an impaired upregulation of collagen I and II in fracture callus and a negative influence on osteoclast function and mechanical strength and maturation of fracture callus^9^. Neurotransmitters were therefore postulated to play a central role in the interaction between the nerves and the osseous system^8,14^, and especially substance P seems to have a significant involvement in bone metabolism, formation, and resorption, as well as in the osteogenic activity of bone marrow stromal cells and osteogenic cell lines^15–23^. However, the relevance of specific neurotransmitters remains unclear in these previous studies. and the specific effects of Substance P on the process of fracture healing have not been elucidated. Therefore, the aim of this study was to investigate the effects of specific Substance P-receptor blockage on gene expression, callus formation, and the mechanical strength of healing fractures in an isolated femur fracture model in rats. We hypothesize that specific substance P receptor blockage impairs mechanical strength of healing fractures, decreases gene-expression in the early fracture healing stages and reduces callus quantity and quality.

## Results

### Animal Mortality

At the beginning of the experiments, all animals were in good general health, according to the ‘score sheet’ and ‘Body condition scoring’ described in the methods section. No rats died during our study due to impacts of the operative procedure or its sequelae. In the course of the experiments, 2 rats (2.78%, planned for histological analysis) had to be excluded according to our exclusion criteria; one animal had to be removed due to wound dehiscence and the other animal due to anaesthetic complications. In total, this left 70 rats (97.22%) for inclusion in the final analyses.

### Gene Expression Analysis (Collagen 1A2- and 2A1- and Osteocalcin-mRNA)

**Figure 1:**
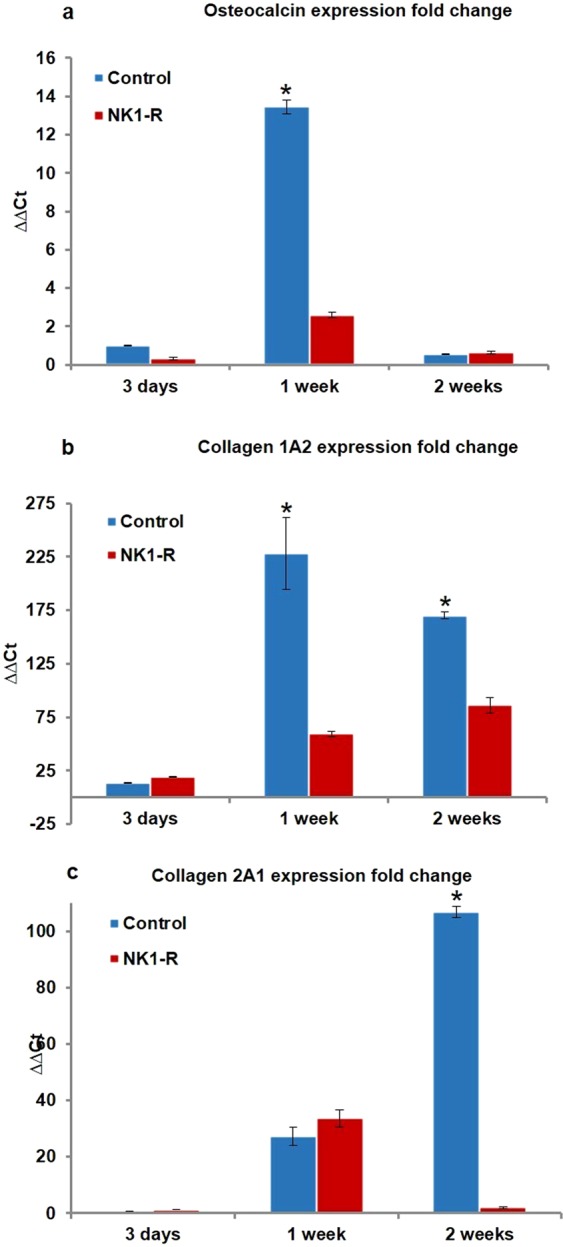
Comparison of fold changes in gene expression. The fold changes of osteocalcin (**a**), collagen 1A2 (**b**), and collagen 2A1 (**c**) mRNA expression were determined by the reverse transcription polymerase chain reaction (RT-PCR) at 3 days, 1 week, and 2 weeks after fracture induction.

Osteocalcin expression was significantly impaired at 1 week in the NK1-R group compared to the Control group (2.6 ± 0.1 fold vs. 13.5 ± 0.4 fold, p = 0.0002 for α = 0.05). Collagen 1A2 also showed a significant depression of expression at 1 week (59.5 ± 2.5 fold vs. 228.2 ± 33.45 fold, p = 0.00148 for α = 0.05), as well as at the 2-week time point (86.2 ± 7.3 fold vs. 170.3 ± 3.2 fold, p = 0.00028 for α = 0.05). Collagen 2A1 expression showed a significant reduction only after 2 weeks (1.9 ± 0.4 fold vs. 106.9 ± 2.1 fold, p = 0.000041 for α = 0.05) (Fig. 1).

### Histological Analysis

**Figure 2:**
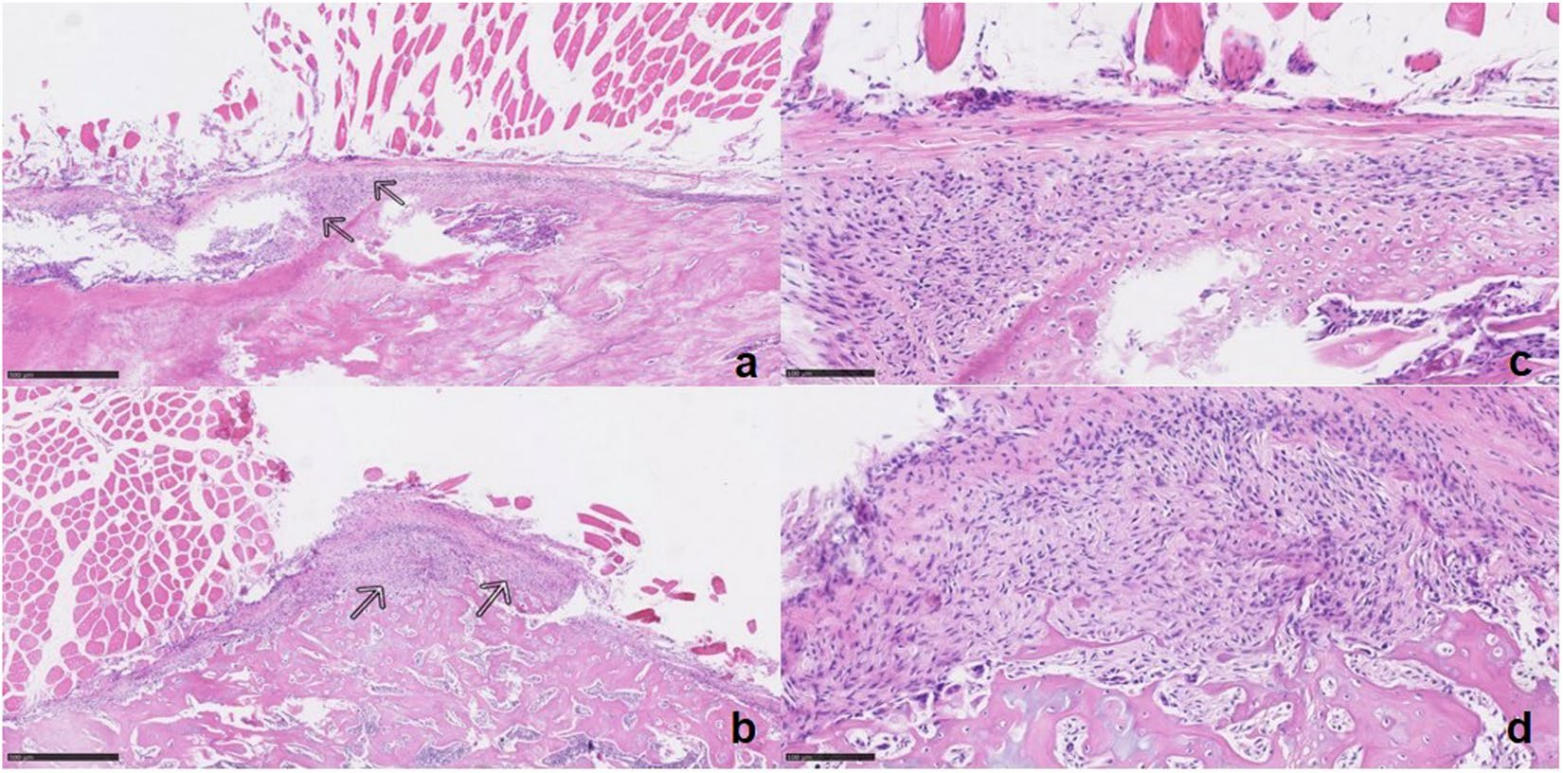
Microscopic imaging of callus tissue. Callus tissue (arrows) between muscle cells (above) and bone (below) in control group (**a**; calibration = 500 µm) and NK1-R group (**b**; calibration = 500 µm) in 5× magnification using haematoxylin-eosin staining. Callus tissue in control group (**c**; calibration = 100 µm) and NK1-R group (**d**; calibration = 100 µm) in 20× magnification using haematoxylin-eosin staining.

The haematoxylin-eosin staining 4 weeks after fracture did not reveal any significant differences in terms of vascularity or angiogenesis. The number and diameter of vessels were similar in both groups. We performed three microscopical measurements in each animal, but neither in trabecular diameter nor in callus diameter significant differences between both study groups were found (Fig. 2).

### Micro-CT Scanning

**Figure 3:**
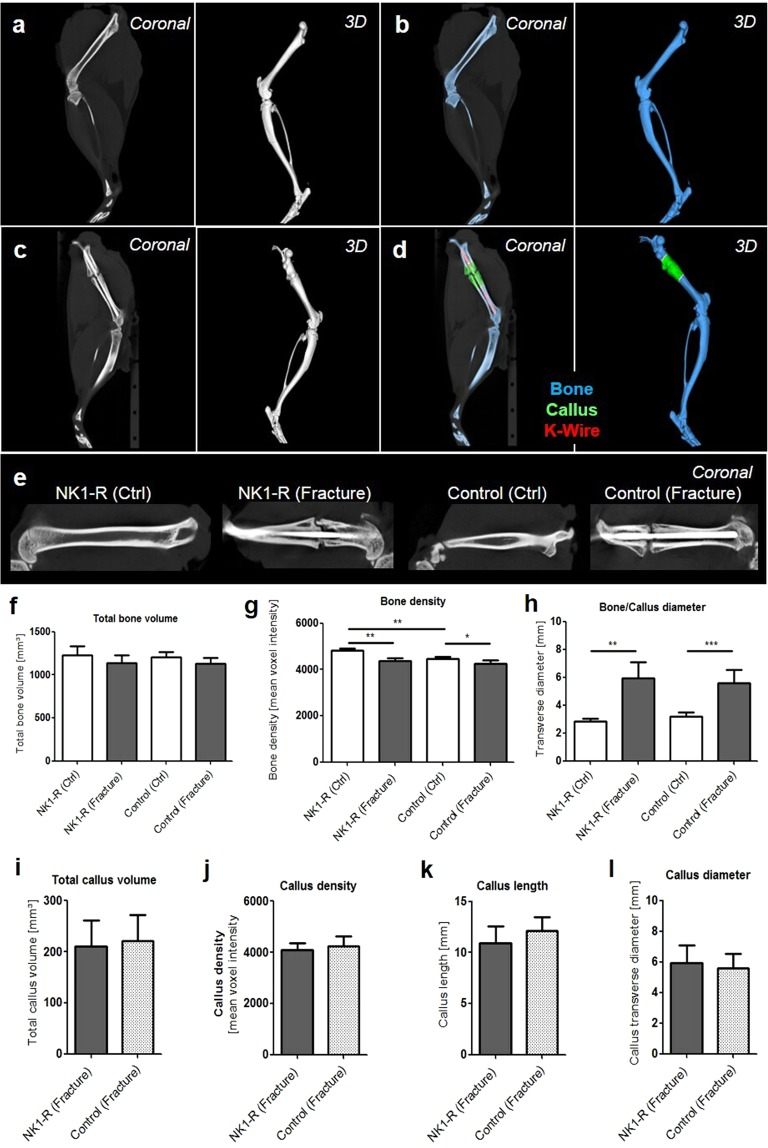
Micro-CT imaging shows the process of ossification and remodelling of the fracture site in the control group and NK1-R group. Micro-CT-imaging of the unfractured control left femur as 2D cross-sectional image in sagittal plane as well as 3D volume renderings resulting in a spatial resolution of 35 µm voxel side length before (**a**) and after segmentation of the bone (blue) (**b**); micro-CT-imaging of the fractured right femur as 2D cross-sectional image in sagittal plane as well as 3D volume renderings resulting in a spatial resolution of 35 µm voxel side length before (**c**) and after segmentation of the bone (blue), callus (green) and K-Wire (red) (**d**). 2D cross-sectional images in sagittal plane for one unfractured and fractured side of both study groups (yellow line as example for callus length and diameter) (**e**). Micro-CT-based quantification of the total bone volume (**f**) and bone density (**g**) of the whole femur in both the fractured side and the control side of both study groups. Diameter at the fracture side compared to the diameter at the correspondence level of the unfractured side (**h**). Micro-CT-based quantification of the total callus volume **(i**), callus density (**j**), callus length (**k**) and callus diameter (**l**) in the fractured side of both study groups. Significance: *p < 0.05, **p < 0.01, ***p < 0.005.

The micro-computed tomography analyses conducted six weeks after fracture induction showed an almost completed process of ossification and remodelling of the fracture site in the Control group, whereas the healing process in the NK1-R group was still in progress, based on the visual analysis of the µCT-scanning images, preceding the quantitative computer tomography analyses. In the quantitative computer tomography analyses, six weeks after healing, the total bone volume did not significantly differ between the fractured side with callus formation and the unfractured side without callus formation in both study groups. This implies that the comparability of the two study groups is warranted. The bone density was significantly lower for the fractured femur compared to the femur on the unfractured side in both groups (NK1-R group, p < 0.01; Control group, p < 0.05). Furthermore, a significant difference was noted in the transverse diameter between the fractured side (callus) and the unfractured side in both groups (NK1-R group, p < 0.01; Control group, p < 0.005). No significant differences were observed between the Control and the NK1-R groups (Fig. 3).

The callus formation analysis did not reveal any significant differences between the two groups in terms of the volume, diameter, length, or density of the callus (Fig. 3).

### Biomechanical tests

**Figure 4:**
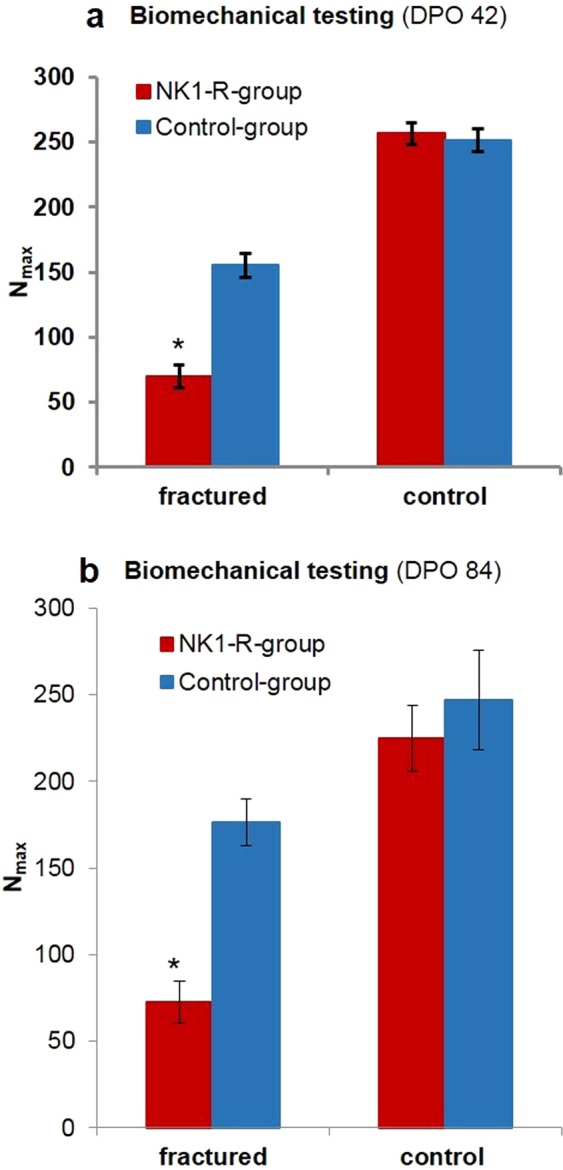
Biomechanical testing of the femora. Significant decrease in maximal load to failure (N_max_) of the femora in the NK1-R group at (**a**) 6 weeks (*p = 0.029) and (**b**) 3 months (*p = 0.01) after fracture induction.

Both study groups demonstrated a decreased load to failure of the fractured femora compared to the unfractured side at 6 weeks and 3 months after fracture. By contrast, biomechanical analyses at 6 weeks (Control group 251.84 ± 8.87 N vs. NK1-R group 254.48 ± 9.95 N) and 3 months (Control group 247.00 ± 28.66 N and NK1-R group 224.67 ± 19.10 N) after fracture showed no significant differences in the load to failure for the unfractured side in both groups. The fractured side showed a significant (p = 0.029) difference in the load to failure between the NK1-R group (69.78 ± 8.51 N) and the Control group (155.45 ± 9.32 N) after 6 weeks. This difference was even greater at 3 months after fracture (NK1-R group: 72.50 ± 12.09 N vs. Control group: 176.33 ± 13.44 N, p = 0.010) (Fig. 4).

### Summary of study results

Our main results can be summarized as follows:

1. Blocking of the NK1-receptor for Substance P was associated with a reduced expression of different osteogenic proteins (osteocalcin, collagen 1A2, and collagen 2A1) in the early phase (the first two weeks) of fracture healing.
2. NK1-receptor blocking impaired the normal improvement in biomechanical strength of the bone in the late phase (6 weeks and 3 months) after fracture.
3. Since the quantitative callus extensions showed no significant differences, but the biomechanical strength of the healing bone was impaired, NK1-receptor blocking decreased the quality of the callus formed.

## Discussion

Fracture healing is well recognized as a complex process that is still not completely understood. However, the relationship between fracture healing and neurologic injury is well described and implies an influence of neurological neurotransmitters^12,13^. Among the various neurotransmitters, Substance P in particular is believed to play a central role in bone metabolism. In this context, Substance P has been shown to stimulate bone formation by osteoblastic cells through interaction with the NK1-receptor^24^, whereas blockage of the NK1-receptor enhanced widespread osteoporotic processes^18^. In the present study, our aim was to investigate what influence blocking the NK1-receptor for substance P might have on fracture healing.

The important role of Substance P in bone metabolism was especially supported in the present study by the demonstration, for the first time, that selective blockage of Substance P activity by a NK1-receptor antagonist impairs the gene expression, biomechanical strength, and callus quality of a healing fracture.

The decreased gene expression observed in the present study for osteocalcin, collagen 1A2, and collagen 2A1 in the early phase of callus formation after Substance P receptor blockade underscores the relevance of Substance P in fracture healing. These results are in line with previous RT-PCR studies that showed a similarly impaired expression of osteocalcin and collagen during fracture healing in tachykinin-deficient mice^15^, in sensory denervated rats^9^, and in rat osteoblastic cells^24^.

However, beside this role in early fracture healing, Substance P seems also to have relevance for the later stages of fracture healing and remodelling of bone. In this context, we were able to show an association between NK1-receptor blockade in the first two weeks of fracture healing and a significant decrease in bone strength at 6 weeks and even at 3 months after fracture. In previous studies, Niedermair and colleagues showed that tachykinin-1-deficient mice (Substance P knockout mice) have a reduced mechanical strength of callus after 3 weeks of fracture healing, while Apel and colleagues showed that sensory denervated rats have a reduced mechanical strength of callus after 6 weeks of fracture healing^9,15^. However, none of these studies analysed the biomechanical strength at the end of the remodelling phase, as we did after 3 months. If these effects of NK1-receptor blockage are exerted in the remodelling phase or are a result based on the earlier effects of NK1-receptor blockage on fracture healing has to be explored in future studies.

Contrary to the results presented by Apel and colleagues, our findings did not indicate any significant histological differences in trabecular or callus diameter. We also did not find any significant radiological differences in quantitative callus dimensions in the µCT-analyses between our two study groups, although Apel and colleagues did report significant radiological differences. Our results agree with the observations of Niedermair and colleagues, who found no radiological differences in callus dimensions between tachykinin-deficient mice and a control group^15^. These comparable quantitative callus dimensions between our study groups, in combination with the decrease in biomechanical strength of the bone in the NK1-R group, can only occur if the blockage of the NK1-receptor for Substance P impairs the quality of the soft and hard callus formed, although we could not substantiate this presumption with histological proof. In this context, the quality of callus is determined by the biomechanical strength of the callus and later of the remodelled bone, because the objective of fracture healing is to restore the original strength of the bone. Since Apel and colleagues performed a denervation that blocked both CGRP and Substance P, this might indicate that CGRP has a greater influence on the amount of callus formed, while Substance P regulates the strength and quality of callus.

The findings presented here demonstrate the indispensability of Substance P for normal and appropriate callus formation and fracture healing. Nevertheless, clarifying the exact role of Substance P in fracture healing and bone formation will require further research. Additional research is also required to assess previously postulated mechanisms of action. For instance, Niedermair and colleagues proposed crucial trophic effects of neurotransmitters on bone healing via an endogenous callus signalling loop in which chondrocytes producing Substance P and its NK1-receptor play an important role^15^. They consider that the absence of Substance P results in a net decrease in bone formation, leading to the observed decrease in the quality of the callus.

Another interesting theory introduced by Davis and colleagues assumed that bone morphogenetic proteins (BMPs) released at the fracture site enter peripheral neurons through the damaged blood–nerve barrier. There, they induce a neuroinflammation with a release of substance P, as well as a subsequent release of osteoprogenitor cells in cases of heterotopic ossifications^25^. The results of our study could also point to a possible lead for further research into enhanced fracture healing in patients with concomitant TBI^13,26–28^, because Substance P is released early following acute injury to the CNS. This promotes a neurogenic inflammatory response that is characterized by an increase in the permeability of the blood–brain barrier^29^

Our study also has some limitations. As our findings were derived from an animal model, they are not directly translatable to humans. We chose a rat model because the blockage of a neurotransmitter can only be performed in laboratory animals and because adequate blockage of the substance P-receptor with the NK1-receptor antagonist SR140333 has been previously demonstrated in this species^30^. Furthermore, the fracture model in rats is well documented in the existing literature, and the influence of NK1-receptor blockage on the complex, multifactorial process of fracture healing cannot be simulated in an *in vitro* experiment.

Moreover, the volume measurements conducted using µCT analyses might be inaccurate due to obscuring by the intramedullary Kirschner wire, which in turn would reduce the volume measurement in the fractured bones. Therefore, the segmentation of the bone, the K-wire and the fracture callus regions was performed with a very advanced interactive method (Software Imalytics Preclinical) to minimize the error range. Even if we had removed the K-wire shortly before the µCT analysis, this removal would have left an empty space, which would not be filled with new callus tissue and therefore would probably lead to the same results. Conversely, our histology findings concerning trabecular and callus dimensions conform to our µCT findings, indicating that these results are probably reliable. Another possible limitation, was that we did not follow up the blocking capacity of SR130444 after finishing the administration after two weeks of fracture healing. We assume that the effect of SR130444 is depleted >24 hours after application, but we did not verified it with immuno-histological tests.

In conclusion, our study findings show that blocking the NK1-receptor for substance P suppresses gene expression of important proteins in the early phases of fracture healing, decreases the quality of callus formed, and lessens the biomechanical strength of bone in the late phases of fracture healing. Therefore, based on our results, further research should be focused on the exact mechanism of action of Substance P in fracture healing to provide a better understanding of its potential therapeutic relevance in cases of disturbed fracture healing.

## Methods

All methods were performed with the approval of the institutional animal committee and of the regulating authority (LANUV) North Rhine-Westphalia, Recklinghausen, Germany (AZ 84-02.04.2015.A078). All animal experiments were performed in accordance with the guidelines and regulations of the Federation of European Laboratory Animal Science Associations (FELASA) and the German Society of Laboratory Animals (GV-SOLAS).

### Housing

Our study cohort consisted of 72 adult, female Sprague-Dawley rats, weighing approximately 250 g, obtained from Harlan Industries (Indianapolis, Indiana, USA). The animals were housed and the experiments were performed in facility approved by the Federation of European Laboratory Animal Science Associations (FELASA) and the German Society of Laboratory Animals (GV-SOLAS). The animals were housed under conditions of controlled temperature (20 ± 2 °C) and air humidity (45–65%), with a 12 h light-darkness cycle and a light intensity of <200 lux. Food and water were offered ad libitum. Prior to study inclusion, all animals were kept in groups for one week in the laboratory premises to allow acclimatization. Throughout the entire experiment, all animals underwent physical examinations according to a ‘score sheet’ documentation and the ‘Body condition scoring’ according to Hickman^31^ to obtain the general health of the animals.

Because of the protective effects of female hormones in cases of inflammatory stimuli, all animals were confirmed to be in the same ‘metestrus’ phase of the menstrual cycle, as this phase is characterized by low estrogen and progesterone levels^14,32^. The menstrual cycle phase was identified by the assessment of vaginal swabs according to Marcondes and colleagues^33^.

### Experimental design and power analysis

**Figure 5:**
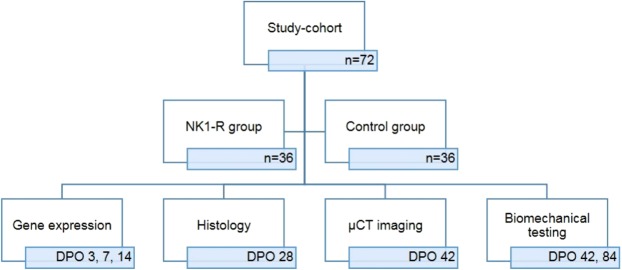
Subgroup division and time points of analyses. Subdivision of study cohort in two study groups (NK1-R group and Control group) and subsequent division of animals according to performed analysis at different time points. Every analysis is performed in 6 animals of both study groups at every time point. At DPO (days post-operative) 42 both µCT imaging and biomechanical testing was performed in the same animals.

In all animals an intramedullary pin was inserted and a standardized femoral fracture was induced. The animals were then randomly assigned to 2 groups (36 rats/group), according to their order number (odd or even). The first group underwent a selective blockage of the NK1-receptor (NK1-R group). The second group served as the control group and only received vehicle (Control group). Depending on the experimental subgroup to which an animal was randomly assigned, the femur was harvested and the following analyses were executed at different time points: gene expression analysis, histological analyses, micro-CT scanning, and biomechanical testing (Fig. 5).

Our primary outcome parameter was the load to failure of the rat femora; therefore, our power analysis was based on an average load to failure of 131.3 ± 4.9 N for normal rat femora, as described by previous studies^9,34^. Our calculation indicated that a minimum of 4 rats per group was needed to achieve a 95% power for detecting a difference of 10 N with α = 0.05. Therefore, a sample size of six animals per group was chosen to compensate for any potential loss of animals. The power analysis was performed with G*Power. Secondary outcome parameters were fold change in gene expression of osteocalcin and collagen 1A2 and 2A1 and callus-volume, -density and -diameter in micro-CT-analysis.

Reasons for excluding animals were: death from anaesthetic complications, open fracture, comminuted fracture, implant failure, wound dehiscence or infection, gross technical failure, inadequate RNA for analysis, and unintended displacement of the femur during biomechanical testing.

### Anaesthesia and pain management

Thirty minutes preoperatively, the animals received 0.03–0.05 mg/kg buprenorphine hydrochloride s.c. as pain medication. The operative procedures were performed under general anaesthesia induced with ketamine (100 mg/kg i.p.) and xylazine (2%; 10 mg/kg i.p.) and if necessary, extended with 2–2.5 Vol.% isoflurane inhalation. The toe pinch reflex was used to assure adequate anaesthesia. Post-operative analgesia was assured with buprenorphine hydrochloride (0.03–0.05 mg/kg s.c.) every 6 h for the first 24–48 h. Subsequently, buprenorphine hydrochloride was given twice daily during the first 3 weeks. Furthermore, in the first postoperative week, the drinking water was supplemented with metamizole (1 ml/300 ml). The animals were evaluated postoperatively three times per day for signs of acute deterioration.

### Standardized femoral fracture

After anaesthesia induction, the animals were placed on a heated pad (37 °C) and their eyes were covered with moistening ointment. The right rear leg was shaved, disinfected, and draped. Then, a para-patellar incision was made, the patella was everted laterally and a 1.0 mm stainless-steel intramedullary Kirschner wire was inserted in a retrograde manner. Its placement was confirmed by fluoroscopy. The K-wire was cut flush with the intercondylar notch and the proximal end was bent over the greater trochanter, cut, and hidden subcutaneously. The patella was repositioned and the wounds were closed in layers. Fracture induction was performed with a blunt guillotine according to the method of Bonnarens and Einhorn^35^. A fluoroscopic evaluation of the fracture site was performed.

### Substance P receptor blockage

Substance P activity was blocked through selective blockage of the neurokinin-1-tachykinin-receptor (NK1-R) by subcutaneous administration of SR140333 once daily for the first 14 days, beginning 30 min preoperatively. This period was chosen because it is the most vulnerable period in the fracture healing process in rats in terms of gene expression and the influence of cytokines, chemokines, growth factors, etc^36,37^. SR140333 is a non-peptide antagonist of tachykinin NK1 receptors that potently, selectively, and competitively inhibits substance P binding to NK1 receptors^38^. SR140333 was administered at a concentration of 1 mg/kg dissolved in a vehicle of 10 μl DMSO (dimethyl sulfoxide) and 0.2 ml of sterile water^30,39^. The animals of the control group received the same amount of vehicle for the same period.

### Gene expression

The up-regulation of most genes associated with fracture healing in rats takes place in the first two weeks after fracture induction^36,37^; therefore, gene expression was measured at 3, 7, and 14 days post-operative (DPO).

At each time point, 6 animals from each group (NK1-R group and control group) were euthanized with an overdose isoflurane and subsequent cervical dislocation. The intramedullary K-wire was removed and bone and callus at 0.5 cm on each side of the fracture was harvested, frozen in liquid nitrogen, pulverized, and used for RNA extraction. The fold changes of osteocalcin, collagen 1A2, and collagen 2A1 mRNA expression were determined by the reverse transcription polymerase chain reaction (RT-PCR). The cDNA was transcribed from total RNA using a Maxima H Minus cDNA synthesis kit (Thermo Scientific, US). Real-time quantitative RT-PCR was performed with Power SYBR Green Master Mix (Applied Biosystems, US) using the StepOnePlus™ Real-Time PCR System (Invitrogen, CA, USA). The 2^−ΔΔC^T method (a method to analyse the relative changes in gene expression from real-time qPCR experiments) was used to calculate gene expression with peptidylprolyl cis-trans isomerase (PPIA) as an internal housekeeping gene reference. The NK1-R and Control groups were compared for their expression of collagen-1A2, collagen-2A1, and osteocalcin mRNA.

### Histological analysis

At DPO 28, 10 animals underwent histological analysis (6 of the αNK1-R group and 4 of the Control group, due to drop out of 2 animals, see *Results* section). The animals were euthanized, the Kirschner wire was removed, and the femora were harvested and fixed in 4% neutral buffered formalin for 48 hours. Decalcification was performed with ethylenediaminetetraacetic acid (EDTA) and dehydration with alcohol. The tissue was embedded in paraffin and 10μm sagittal sections of the callus area and the surrounding soft tissues were cut. The sections were stained with haematoxylin eosin (HE) and assessed with an Olympus BHS System Microscope. Images were viewed at 40× and 200× magnification, and measurements of the bone were performed.

### Micro-computed tomography (μCT)

Micro-CT imaging was obtained at DPO 42 in 6 euthanized animals from each group using a dual-energy gantry-based flat-panel microcomputed tomography scanner (TomoScope 30 s Duo, CT Imaging, Erlangen, Germany). The dual-energy X-ray tubes of the μCT were operated at voltages of 40 and 65 kV, with currents of 1.0 and 0.5 mA, respectively. Coverage of the entire leg of the rats was achieved by performing three sub-scans; each sub-scan acquired 720 projections with 1,032 × 1,012 pixels during one full rotation, with durations of 90 s. After acquisition, volumetric data sets were reconstructed using a modified Feldkamp algorithm with a smooth kernel at an isotropic voxel size of 35 μm. The bone, K-wire, and fracture callus regions were segmented using an automated segmentation method with interactive correction of segmentation errors (Software Imalytics Preclinical^40^). (Fig. 2) The total bone volume, callus volume, bone density, callus density, and transverse diameter of the fracture callus were analysed quantitatively. The mid-part of the femoral shaft of the unfractured side of the animals from the Control group were taken for reference.

### Biomechanical testing

Biomechanical testing was performed at two time points in the healing process to analyse the strength of the callus and/or newly formed bone. The first test was performed at DPO 42 in 6 animals of both groups; the second test was performed at DPO 84 in 6 animals of both groups. These time points were chosen because normal callus formation is completed after 6 weeks and the remodelling process is advanced or completed after 3 months. The fractured right as well as the unfractured left femur were tested in all animals. After euthanizing the animals and removing the K-wires, both ends of the femora were embedded, closely to the callus/fracture site in a two-component resin (Technovit® 3040 powder + Technovit® Universal Liquid)^41,42^, which quickly hardens at low temperature. The embedded femora were tested in the biomechanical testing device (Retroline from Zwick Roell AG, Germany), over two cardan yokes, by which the force was conducted perpendicular to the femur axis. The biomechanical traction test was performed with a traction rate of 1 mm/s = 0.1 N/s and a measurement interval of 0.1 s. Digital set-up and control was performed using TestXpert II software (Zwick Roell AG, Germany), which enabled real-time measurement of the traction force. The obtained parameters were used for the calculation of average load to failure in Newton (N).

### Data analysis

The data were analysed using the Statistical Package for the Social Sciences (SPSS; version 22; IBM Inc., Somers, NY, USA) and GraphPad Prism 5.0 (San Diego, CA, USA). Continuous data are presented as mean ± standard deviation, while incidences are presented as counts and percentages. Differences between the groups were evaluated with a two-tailed unpaired Student’s t-test and with analysis of variance (ANOVA with post hoc Tukey) for continuous data. In general, Pearson’s χ2-test was used for categorical values. The gene expression and biomechanics data were analysed with the Mann–Whitney U test as a non-parametrical test. Potential statistical associations were evaluated with Pearson’s correlation. In general, a two sided p-value < 0.05 was considered statistically significant.

### Proofreading

Scribendi Inc. (Chatham, Ontario N7M 0N3 Canada) performed the proofreading of our manuscript.

## Data Availability

The datasets generated during and/or analysed during the current study are available from the corresponding author on reasonable request.
